# The Efficacy of Green Synthesized Nanosilver in Reducing the Incidence of Post-Harvest Apple Fruit Brown Rot

**DOI:** 10.3390/jof7060473

**Published:** 2021-06-10

**Authors:** Adel Kamel Madbouly

**Affiliations:** Microbiology Department, Faculty of Science, University of Ain Shams, Cairo 02, Egypt; adelkamelmadbouly@yahoo.com or adel_ramadan@sci.asu.edu.eg

**Keywords:** antifungal potential, in vivo control, *Malus pumila* Mill., *M. fructigena*, nanopreservative

## Abstract

This study aimed to green synthesize nanosilver (AgNPs) using black tea extract and use it as a nanopreservative to increase the shelf life of stored apple fruits. Ultraviolet visible absorption (UV–vis) analysis of AgNPs recorded two λ max values at 260 and 452 nm. Transmission electron microscope and dynamic light scattering analyses showed that AgNPs are spherical in shape and have an average size of 20 and 170.6 nm, respectively, with a zeta potential of −20.06 mV. An in vitro assay confirmed the antifungal potential of AgNPs against *M. fructigena* when applied at 200 mg/L and preincubated for 4 days, reducing the radial growth by 96.1%. At the same dose and preincubation period, AgNPs caused a significant reduction in the diameter and fresh weight of brown rotted lesions in apple fruits artificially coinoculated with the pathogen by 77.4% and 84.4%, respectively. AgNPs caused the leakage of proteins and DNA from *M. fructigena* conidia and did not express cytotoxicity against the human HaCaT cell lines. Accordingly, green synthesized AgNPs are eco-friendly and economical and do not pose harm to human health; thus, they could be used as an effective nanopreservative in apple fruit stores to reduce the incidence of brown rot disease.

## 1. Introduction

Approximately 20–25% of the harvested fruits and vegetables worldwide show symptoms of rotting due to fungal infections; thus, controlling these diseases is highly recommended by phytopathologists [1]. Fresh fruits play major roles in our healthy daily diet as they contain several nutritionally active substances [2]. A previous study conducted by [3] has revealed that apple flesh (*Malus pumila* Mill.) and its skin have dietary fibers that clean the intestinal tract through the removal of harmful substances and reduce the risk of gallstone diseases. *Monilinia fructigena* (Honey) is a fungal pathogen that causes pre- and postharvest rots in pome fruits. Ref. [1] have reported that almost all pre- and postharvest fungal infection control methods have been based on the use of synthetic fungicides. A study conducted by [4] has highlighted that these chemicals are harmful to human health, expensive, and not ecofriendly. Accordingly, phyto-pathologists are looking for alternative methods to control most of these postharvest fungal diseases [5]. 

Ref. [6] have reported that nanobiotechnology is a rapidly growing area that has various applications in science and technology, in order to produce novel nanosize particles about 1–100 nm in diameter. AgNPs are nanoparticles (NPs) whose unique chemical, physical, and biological properties have attracted considerable attention. NPs are synthesized through several physical, chemical, and biological methods, which expand the NPs’ antimicrobial activity depending on their size, shape, and chemical composition [7]. Most of the commonly used methods to synthesize NPs are associated with several disadvantages. However, [8] recently reported that green synthesis of AgNPs using plant extracts produces NPs that have different morphological characteristics, including sizes, stability, and electronegativity, which contribute to their bacteriostatic and fungistatic inhibitory potentials at significantly low concentrations. Black tea extract used in the current study for the synthesis of AgNPs is a brain stimulating beverage that is extracted from the leaves of the tea plant (*Camellia sinensis*), which is widely cultivated in several Asian countries [9]. 

The use of NPs to promote plant growth and control microbial diseases is a well-developed technique, as proposed by [10]. Later, Ref. [11] revealed that AgNPs are used as an effective antimicrobial agent. A previous study conducted by [12] documented the antimicrobial potential of AgNPs against several pathogenic microbes, including viruses, fungi, and bacteria. Accordingly, as a promising alternative to the chemical fungicides, AgNPs are currently used in the management of many fungal crop diseases [13]. The objectives of this work were to green synthesize AgNPs using black tea extract and to use these AgNPs in preventing the postharvest decay of apple fruits caused by *M. fructigena*. Accordingly, the current findings will increase the shelf life of the stored apple fruits through the use of AgNPs as a potent, economic, and ecofriendly nanopreservative.

## 2. Materials and Methods

### 2.1. Sample Collection

About 20 samples of diseased apple fruits cv. (Golden delicious) showing typical symptoms of brown rot were collected from the El-Tawfikia street markets, Cairo governorate, Egypt. These fruits were collected in sterile polyethylene bags and then immediately delivered to the laboratory where the fungal pathogen was isolated.

### 2.2. Isolation and Identification of the Fungal Causal Agent of Apple Brown Rot

The brown rot fungal pathogen was isolated from the diseased apple fruits, and then molecularly identified by sequencing of the ribosomal DNA-ITS regions using PCR according to the previous study of [14]. An accession number of MH862738.1 was assigned to this isolate of *M. fructigena*.

### 2.3. Green Synthesis of AgNPs Using Crude Tea Extract

According to [15], black tea (10 g) was immersed in 100 mL of DDS in a 250 mL conical flask, and the mixture was heated at 60 °C for 5 min. The crude tea extract was filtered through Whatman no. 1 filter paper, and then 20 mL of the extract was combined with 80 mL of 1 mmol/L silver nitrate (AgNO_3_) solution (Merck; Darmstadt, Germany). The solution was incubated under dark conditions in a water bath at 60 °C for 30 min. Change in the color of the solution from yellow to brown indicated the formation of AgNPs. The AgNPs suspension was centrifuged at 10,000 g for 10 min, and then the supernatant was disposed of. The precipitate was resuspended in DW and then centrifuged again three times. The separated AgNPs were dried in an oven at 50 °C for 12 h.

### 2.4. Characterization of the AgNPs

#### 2.4.1. The Ultraviolet Visible Absorption Spectra

The AgNPs were characterized using ultraviolet visible (UV–vis) absorption spectra through a PerkinElmer LAMBDA, 35 UV–vis spectrophotometer (JEOL, Peabody, MA, USA). The UV–vis spectrum of the samples was recorded in the range of 300–800 nm according to [16]. 

#### 2.4.2. The Transmission Electron Microscope (TEM)

Transmission electron microscope (TEM) (CARL ZEISS, Jena, Germany) analysis was used to determine the shape and size of the AgNPs. The measurement was carried out using a HITACHI H-800 operating at 200 kV in reference to [17]. 

#### 2.4.3. The Fourier Transform Infrared (FTIR) Analysis

According to [18], FTIR measurements were carried out on the AgNPs to identify the possible presence of bioactive molecules in the crude tea extract that were responsible for reducing the silver ions. The spectrum was recorded using three replicates through a wavelength interval of 400–450 cm^−1^ using a resolution of 4 cm^−1^. Molecular analysis of the AgNPs sample was carried out using an FTIR spectroscopy Perkin-Elmer-1600 (Waltham, MA, USA).

#### 2.4.4. The Dynamic Light Scattering (DLS) and Zeta Potential

The hydrodynamic diameter and zeta potential (ZP) of the NPs were analyzed on a Zeta-sizer Nano ZS ZEN3600 from Malvern Instruments Ltd. (Worcestershire, UK). The results were expressed as mean ± standard deviation (SD) in three replicates in reference to [19]. 

### 2.5. Detection of the In Vitro Antifungal Potency of AgNPs against M. fructigena

#### 2.5.1. The Poisoned Food Technique

The antifungal potential of AgNPs against *M. fructigena* has been studied on PDA (Merck; Darmstadt, Germany), malt extract agar (MEA) (Merck; Darmstadt, Germany), and corn meal agar (CMA) (Merck; Darmstadt, Germany) media using the poisoned food technique in reference to [20]. The AgNPs solutions were prepared with four different concentrations: 50, 100, 150, and 200 mg/L. Each concentration was added aseptically and individually to the three media after autoclaving and then poured into Petri plates. Plates were preincubated at room temperature for four different periods: the first set of plates was preincubated for 0 h, whereas the remaining three sets were preincubated for 1, 2, and 4 days, respectively. After the 4 different preincubation periods, mycelial disks (3 mm each) were cut from the edge of the 7-day-old culture of the pathogen, and then a single disc was placed at the center of each of these plates. The Petri plates lacking AgNPs and those supplemented with Nystatin antifungal (100 mg/L) served as the negative and positive controls, respectively. The plates were then incubated at 28 ± 2 °C for 6–8 days. Three replicate plates were used for each concentration and for each growth medium, and the assay was repeated three times. The percentages of inhibition (%) of radial growth of the fungal discs with respect to the control plates were measured after 8 days according to the following formula of [20]:

Inhibition percentage = R − r/R × 100

where (*R*) is the radial growth of fungal mycelia on the control plate and (*r*) is the radial growth of fungal mycelia on the plate treated with the AgNPs. Results were recorded as means of three replicates.

#### 2.5.2. The Agar Well Diffusion Assay

The in vitro antifungal potency of AgNPs was assessed on a PDA medium using the agar well diffusion assay of [21]. Four different concentrations of AgNPs were prepared: 50, 100 150, and 200 mg/L. Two hundred microliters of the fungal conidial suspension (2.0 × 10^6^ CFU/mL) were aseptically spread on the surface of PDA plates using a sterile glass spreader. Wells of 3 mm in diameter were made aseptically using a sterile cork borer, and then each of these wells was filled individually with 100 µL of each concentration of the tested AgNPs. Wells inoculated with sterile distilled water and those with Nystatin (100 mg/L) served as the controls. The plates were then incubated at 28 ± 2 °C for 7 days. Three replicate plates were used for each concentration, and the assay was repeated three times. After incubation, zones of inhibition (cm) of the fungal growth were measured using a ruler, and the percentages of inhibition (%) of the pathogen growth were recorded. Results were recorded as means of three replicates.

### 2.6. Determination of Minimum Inhibitory Concentration (MIC) of the AgNPs

The broth dilution method of [22] was used to determine the MIC of the AgNPs against *M. fructigena*. Different concentrations of the AgNPs were prepared, including 5, 10, 15, 20, and 25 mg/L. About 1 mL of each concentration was added individually and aseptically to the test tubes containing 1 mL of sterile PDB. All test tubes were then inoculated with 0.1 mL of the fungal conidial suspension (2 × 10^6^ CFU/mL), and then the tubes were incubated at 28 °C. After incubation for 24 h, approximately 50 µL of each tube was placed on a microscope slide and germination of about 100 conidia per slide was observed under a light microscope. When germ tube’s length was double the diameter of the conidium, it was considered to be germinating. The tubes that lacked AgNPs were considered as the controls. Three replicate tubes were made from each concentration of the AgNPs, and the assay was repeated three times.

### 2.7. In Vivo Control of M. fructigena on the Apple Fruits

#### 2.7.1. Preparation of the Apple Fruits and the AgNPs

Apple fruits cv. (Golden delicious) were surface sterilized with 1% NaOCl for 1 min, washed three times with SDW, and then allowed to dry. Three wounds were made on each fruit using a sterile cork borer (5 mm in diameter and 5 mm deep). The dried AgNPs were weighed and then adjusted to four different concentrations in test tubes using sterile distilled water (SDW) at 25, 50, 100, and 200 mg/L, respectively. These solutions were subjected to sonication for 3 min. Three replicate tubes were prepared for each concentration.

#### 2.7.2. Inoculation of the Treated *M. fructigena* into the Wounded Apple Fruits

The *M. fructigena* was grown on PDA Petri plates at 28 °C for 5–7 days, after which its inoculum was adjusted to 2 × 10^6^ CFU/mL using a haemocytometer. The tubes containing 100 µL of each of the four different concentrations of AgNPs were inoculated individually with 100 µL of the pathogen inoculum. Four sets of test tubes were prepared and then incubated individually at 28 °C for 0 h, 1 day, 2 days, and 4 days, respectively. According to the modified method of [23], wounded apple fruits were aseptically and individually inoculated with 200 µL of each set of the treated pathogen inoculum and then allowed to dry for 1 h. Next, all the inoculated fruits were placed separately in surface sterilized plastic plates in a humid chamber and then covered with aluminum foil. After 10 d of incubation at 28 °C, the disease severity was evaluated according to the percentage (%) of reduction of the diameter (cm) and weight (g) of the rotting lesions of the fruits. Wounded fruits inoculated neither with the pathogen nor with any treatment served as the healthy controls, whereas fruits treated only with the pathogen served as the positive controls. Another set of apple fruits was inoculated with 200 µL of the synthetic fungicide (100 mg/L) treated pathogen inoculum to compare with the antifungal potential of the AgNPs. There were six replicate fruits for each treatment, and the assay was repeated three times.

### 2.8. Leakage of Proteins and DNA from Conidia of M. fructigena

As a possible mode of action, AgNPs may inhibit germination of the conidia of *M. fructigena* through causing leakage of their proteins and DNA. In reference to [24], conidial suspension of *M. fructigena* (2 × 10^6^ CFU/mL) was prepared in a saline solution of NaCl (0.9% *w*/*v*) and then incubated with AgNPs for 24 h at 28 °C (at their respective MIC values recorded previously). A conidial suspension lacking AgNPs was used as a control. After incubation, the conidial suspension in each treatment was centrifuged at 4000 rpm for 15 min at 4 °C. The optical density (OD) of the supernatant was measured at 280 and 260 nm using a spectrophotometer (Foss400; Foss Electric A/S, Hillerod, Denmark) to estimate the leakage of proteins and DNA from the conidia, respectively. Three replicates were used for each treatment and the assay was repeated three times.

### 2.9. Detection of Cytotoxicity of AgNPs

#### 2.9.1. Human Cell Cultures

HaCaT (human keratinocyte) and 1BR3 (human skin fibroblast lines) normal cell lines were provided by VACSERA Institute, Dokki, Giza, Egypt. The HaCaT cells were cultured as a monolayer in high glucose Dulbecco’s Modified Eagle’s medium (DMEM; Sigma-Aldrich, Darmstadt, Germany), whereas the 1BR3 cell lines were grown in Eagle’s Minimum Essential medium (EMEM; ATCC). These cell lines were kept at 37 °C in a humidified atmosphere containing 5% of CO_2_.

#### 2.9.2. The Cytotoxicity Assay

In vitro cytotoxicity of AgNPs was tested using a 3-(4,5-dimethylthiazol-2-yl)-2,5-diphenyltetrazolium bromide (MTT) assay, according to the modified method of [25]. The HaCaT and 1BR3 human cell lines were seeded in 96-well plates at a concentration of 1 × 10^4^ cells/well and then allowed to attach. Next, the old medium was replaced with a fresh one containing AgNPs in dimethyl sulfoxide (DMSO) at different concentrations (i.e., 15, 25, and 50 mg/L), and then the loaded cells were incubated at 37 °C for different periods: 24, 48, and 72 h. Both types of cells were treated with the synthetic fungicide (50 mg/L) and then incubated for 72 h as positive control treatments, whereas the control cells were treated with 0.5% DMSO. After incubation, 10 μL of MTT reagent (5 mg/mL) was added into each well. Within 4 h of contact, the mitochondrial reductase was converted and then precipitated the MTT in the form of blue crystals. These crystals were then dissolved in 100 μL of a lysis solution (Sigma-Aldrich, Darmstadt, Germany). The reduced MTT was finally analyzed spectrophotometrically at 570 nm using a microplate reader (Thermo Scientific SkanIt Microplate Reader Software; Waltham, MA, USA). The assay was carried out in triplicates and repeated three times.

### 2.10. Statistical Analysis

Results were analyzed statistically using an SPSS software package for Windows version 19.0 (SPSS Inc., Chicago, IL, USA). Differences in the mean values of parameters (±standard deviation) were tested by one-way analysis of variance (ANOVA) and separated by Turkey’s honestly significant difference test (*p* < 0.05).

## 3. Results

### 3.1. Green Synthesis of the AgNPs

Green synthesis of the AgNPs was carried out using crude tea extract. The formation of AgNPs was detected through a color change of the tea extract solution from yellow to dark brown. The synthesized AgNPs were characterized using several physical assays.

### 3.2. Characterization of the AgNPs

#### 3.2.1. The Ultraviolet Visible (UV–VIS) Absorption Spectra

**Figure 1:**
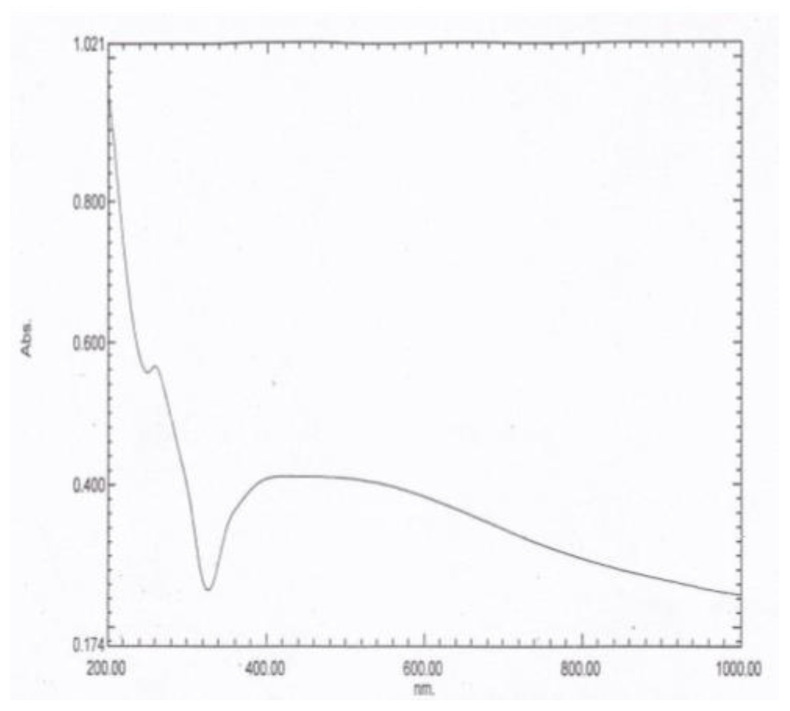
The UV–vis absorption spectrum of the AgNPs showing two characteristic peaks at λ max values of 260 and 452 nm.

The UV–vis absorption spectrum is an essential tool to detect the formation and stabilization of the AgNPs in the aqueous tea extract. The spectral response of the AgNPs was based mainly on their main diameter. Bioreduction of the silver ions to AgNPs was observed successfully using the UV–vis absorbance. When the AgNPs were subjected to UV–vis absorption analysis, two λ max values were obtained at 260 and 452 nm, as demonstrated in Figure 1.

#### 3.2.2. The Transmission Electron Microscope (TEM)

**Figure 2:**
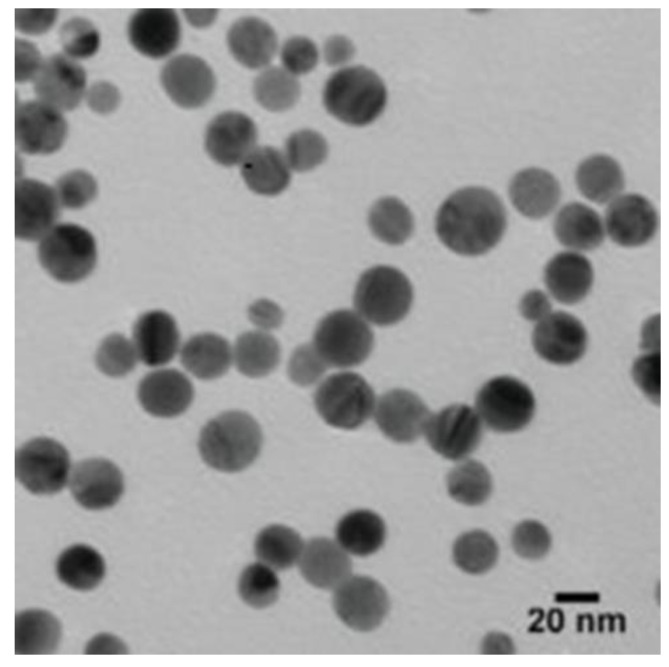
The TEM micrographs demonstrated that the size of the AgNPs was about 20 nm (±5 nm), and they were spherical in shape.

The TEM was used to detect the size and shape of the AgNPs. According to the TEM micrographs, the recorded diameter of the AgNPs was 20 nm (±5 nm), and they were spherical in shape (Figure 2).

#### 3.2.3. The Fourier Transform Infrared (FTIR)

**Figure 3:**
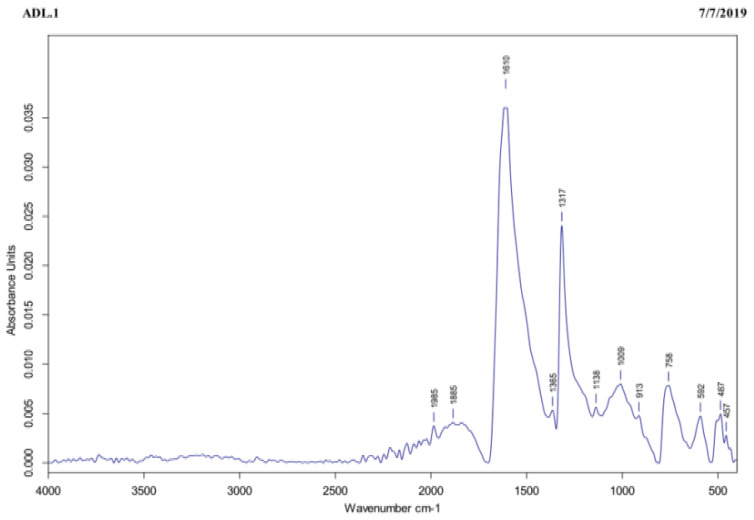
The tea extract subjected to FTIR exhibited characteristic IR-bands appearing at 1610, 1317, 1365, 758, 1009, 592, 487, and 457 cm^−1^, corresponding to several functional groups, such as C=O stretching of amide, C–C aromatic ring, C–O stretching of the amino acid, and hexagonal phase of the AgNPs.

The FTIR assay was carried out to identify the functional groups present in the tea extract that were responsible for reducing the Ag^+^ to AgNPs. In the tea extract, the IR-band appearing at 1610 cm^−1^ represented C=O stretching of amide, as shown in Figure 3. On one hand, the three peaks recorded at 1317, 1365, and 758 cm^−1^ were ascribed to the functional groups and to the C-C aromatic ring present in this extract, whereas the peak observed at 1009 cm^−1^ was referred to C-O stretching of the amino acid. Finally, the peaks recorded at 592, 487, and 457 cm^−1^ were attributed to the hexagonal phase of the AgNPs.

#### 3.2.4. The Dynamic Light Scattering (DLS) and Zeta Potential (ZP)

**Figure 4:**
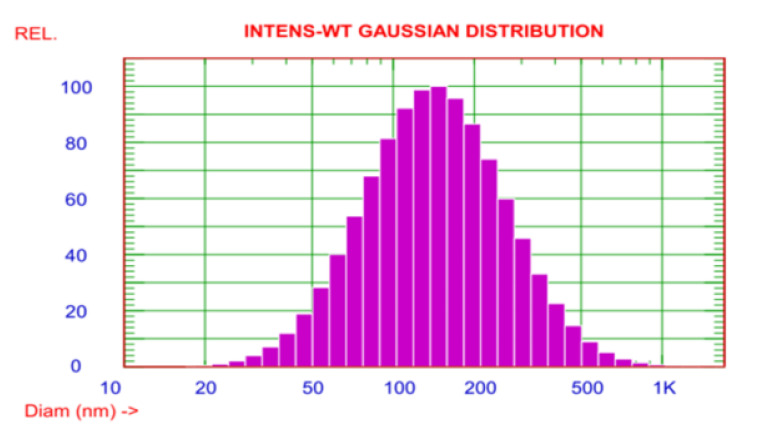
The DLS demonstrated that the size distribution of the AgNPs ranged from 20 to 1000 nm.

To confirm the size of AgNPs, the DLS assay was manipulated. The observed size of these AgNPs ranged from 20–1000 nm. However, the average particle size was about 170.6 nm (Figure 4). The zeta potential of the AgNPs was observed as a sharp peak at −20.06 mV detected by the electrophoretic mobility.

### 3.3. The In Vitro Antifungal Efficacy of the AgNPs

#### 3.3.1. Poisoned Food Technique

To detect the in vitro antifungal potency of the AgNPs against *M. fructigena*, the poisoned food assay was performed. AgNPs caused significant in vitro reduction of the diameter of radial growth of the *M. fructigena* on the PDA medium (Table 1). The percentage of this inhibitory activity increased when increasing the corresponding concentration of the AgNPs (dose-dependent) from 25–200 mg/L, and when increasing the preincubation period (time-dependent) from 0 h to 4 days. Upon using 25 mg/L of the AgNPs and after 0 h, the percentage reduction of radial growth was 17.9%, compared to the control plates. Further increase of the preincubation period to 4 days at the same concentration was accompanied by an appreciable decrease in the percentage of growth by 33.1%. On applying the AgNPs at 50 and 100 mg/L, the percentages of growth decreased significantly by 64.6% and 76.6%, respectively, after 4 days of preincubation. Upon using a high dose of the AgNPs (200 mg/L) and after 0 h of preincubation, a noticeable reduction of the radial growth was observed (67.9%). The recorded reduction in percentage of radial growth upon applying AgNPs at 200 mg/L and after preincubation for 4 days was 96.1%, which was very close to that of the synthetic antifungal, i.e., Nystatin (98.3%), when inoculated at 100 mg/L. Similar results of the inhibitory efficacy of the AgNPs were recorded on using both of the CMA and MEA media (Table 1); however, the decrease in percentage of radial growth was more pronounced on the PDA plates. The means of each of the three replicates were not significant, *p* ≤ 0.05.

#### 3.3.2. Agar Well Diffusion Assay

In order to confirm the in vitro antifungal efficacy of the AgNPs against the pathogen, the agar well diffusion test was carried out. The applied AgNPs caused a pronounced increase in the diameter of inhibition zones of *M. fructigena* growth on the PDA plates. The percentage (%) of increase in inhibition zone diameters was more significant when increasing the corresponding concentrations of the AgNPs (dose-dependent) as presented in Table 2 compared to the synthetic antifungal (61.4%) at 100 mg/L. The means of the three replicates were not significant, *p* ≤ 0.05.

### 3.4. Determination of MIC of the AgNPs

The MIC detects the antifungal potential of AgNPs at its lowest concentrations. On evaluating the efficacy of five different concentrations of the AgNPs (i.e., 5, 10, 15, 20, and 25 mg/L) on inhibiting conidia germination of the pathogen in a PDB medium, the concentration of 15 mg/L was recorded as the MIC of the tested AgNPs.

### 3.5. In Vivo Control of M. fructigena by Coinoculation with AgNPs in Apple Fruit

**Figure 5:**
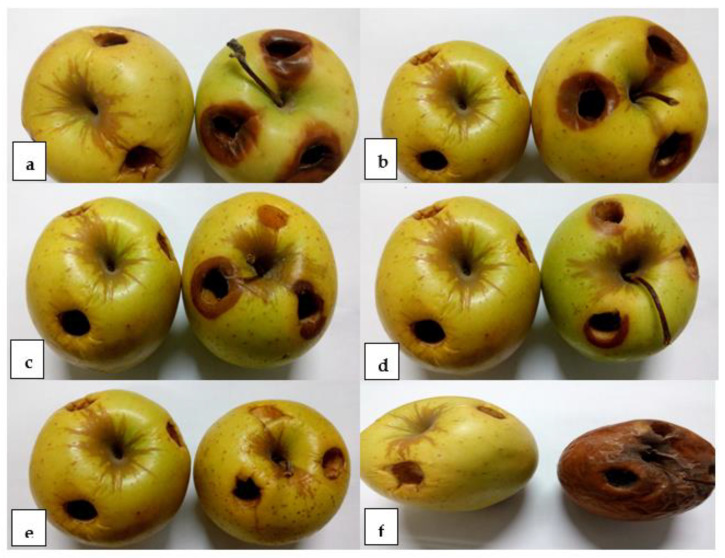
The in vivo antifungal efficacy of different doses of AgNPs (**a**): 25 mg/L; (**b**): 50 mg/L; (**c**): 100 mg/L, and (**d**): 200 mg/L, coinoculated with *M. fructigena* (2 × 10^6^ CFU/mL) into the wounded apple fruits. Significant inhibition of the rot symptoms was observed on the apple fruits treated with the AgNPs, detected through reductions of the diameter and fresh weight of the rotting lesions (concentration dependent), compared to the fruits treated with the synthetic fungicide (**e**), and those fruits treated with *M. fructigena* only (**f**). Six replicate fruits were used for each treatment. In each pair of fruits, the left-hand side fruit is the nontreated control fruit, whereas the right-hand side fruit is the treated one.

**Figure 6:**
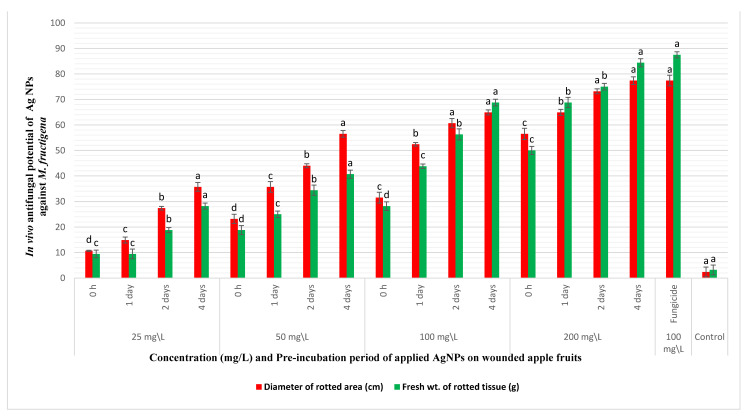
In vivo antifungal potency of different concentrations (25–200 mg/L) and different pre-incubation periods (0 h–4 days) of AgNPs coinoculated with *M. fructigena* (2 × 10^6^ CFU/mL) in wounded apple fruits, and then incubated at 28 °C for 10 days. An appreciable reduction in the percentages (%) of the rot symptoms was recorded, evaluated through reduction in percentages of diameters and fresh weights of the fruits rotting lesions. This inhibitory activity was dose and time dependent, compared to the positive control fruits treated with the synthetic fungicide (100 mg/L) and the nontreated control fruits. Results are means of six replicate fruits of the most accurate assay. Columns with different superscript letters indicate significant differences (*p* ≤ 0.05) based on one-way ANOVA analysis. The error bars represent the ± standard deviations.

The in vivo reduction of brown rot disease incidence on apple fruits was tested through coinoculating the fruits with the pathogen and AgNPs. Nanosilver caused appreciable decrease in the diameters and weights of the rotting lesions on coinoculation with *M. fructigena* into wounded apple fruits, as demonstrated in Figure 5 and Figure 6. The decrease in rotting parameters was dose and time dependent. Coinoculation of the apple fruits with 25 mg/L of AgNPs and the pathogen after 0 h of preincubation caused a reduction in the percentages of the diameters and weights of rotting lesions by 10.8% and 9.45%, respectively, compared to inoculation with the pathogen only. At the same tested concentration, increasing the preincubation periods to 1, 2, and 4 days was accompanied by a pronounced decrease in the percentages of the same tested parameters by (14.9%, 9.45%), (27.4%, 18.9%), and (35.8%, 28.2%), respectively. However, artificial inoculation of the fruits with AgNPs at 50 and 100 mg/L and after a preincubation period of 4 days caused a noticeable reduction in the diameters and weights of the rotting lesions by (56.6%, 40.7%) and (64.9%, 68.9%), respectively. At a high dose of AgNPs (200 mg/L), a pronounced reduction in the percentages of the diameters and weights of the rotting lesions was observed, especially with increasing the preincubation periods throughout 0 h, 1, 2, and 4 days by (56.6%, 50.1%), (64.9%, 68.9%), (73.3%, 75.1%), and (77.4%, 84.4%), respectively. Coinoculation of the apple fruits with *M. fructigena* and 100 mg/L of the synthetic fungicide was accompanied by a noticeable decrease in percentages of the same tested parameters by 77.4% and 87.6%, respectively. The means of the six replicates were not significant, *p* ≤ 0.05.

### 3.6. Leakage of Proteins and DNA from Conidia of the Treated M. fructigena

The probability of leakage of proteins and DNA from the pathogen conidia through treatment with AgNPs was investigated as a possible mode of inhibitory action of the AgNPs. Compared to the controls, the OD of the proteins leaked from *M. fructigena* conidia incubated with AgNPs was 2.51, whereas the OD of the leaked DNA was 1.78. These results confirmed the leakage of proteins and DNA out of the pathogen conidia on treatment with AgNPs.

### 3.7. The Cytotoxicity Assay

This assay was performed to ensure the absence of any cytotoxic activity of the synthesized AgNPs against the normal human cell lines for possible future application as a nanopreservative in apple fruits stores. The percentages of viable human cell lines were detected after incubation with the AgNPs. As shown in Table 3, stimulation of the HaCaT cells was observed as an increase in the cell viability percentage at all the tested concentrations of AgNPs (15, 25, and 50 mg/L), and after all incubation periods (24, 48, and 72 h). Upon using AgNPs at 15, 25, and 50 mg/L and after an incubation period of 72 h, the recorded viability percentages of the HaCaT cells were 100%, 102%, and 105%, respectively, compared to the nontreated control cells. On the other hand, the 1BR3 cells were slightly sensitive to the AgNPs in a dose- and time-dependent manner. The lowest viability percentage (70%) was observed upon incubation of the 1BR3 cells with AgNPs at 50 mg/L and after incubation for 72 h. However, at a low dose of 15 mg/L (MIC of AgNPs) and after 72 h incubation, the 1BR3 cells expressed a high viability percentage (100%), compared to the control. Treatment of both types of cells with the synthetic fungicide caused a significant reduction in the viability percentages of the HaCaT and 1BR3 cells, recording 26 and 21%, respectively.

## 4. Discussion

Isolation of the causal agent of brown rot of apple fruits led to the recovery of a single fungal isolate, identified as *M. fructigena* through amplification of the fungal DNA-ITS region during the previous study conducted by [14]. The synthesis of NPs using plant extract such as black tea is simple, ecofriendly, and economical, carried out at room temperature and in an aqueous environment without organic solvents [26,27]. In this study, AgNPs were prepared through the incubation of the AgNO_3_ solution with black tea extract under dark conditions. The reducing power of the tea extract was attributed to the presence of several phytochemicals including polyphenolic compounds, epicatechin, epicatechin-3-gallate, epigallocatechin, epigallocatechin-3-gallate (EGCG) [28], terpenoids, carboxylic acids, amides, aldehydes, ketones, and flavones [29]. These phytochemicals acted as reducing and capping agents [30]. Additionally, they were also able to stabilize the synthesized AgNPs through interacting with their surfaces [31]. The formation of NPs was observed through a color change in the reaction mixture of the AgNO_3_/tea extract solution, which changed gradually from light yellow to brownish black. This color change was attributed to surface plasma resonance [32], and the reduction of Ag^+^ ions by the tea extract.

The UV–vis absorption spectral analysis of the AgNPs exhibited a gradual decrease, where maximum absorptions were recorded at λ of 260 and 452 nm. This result might be attributed to the blue shift and dependence of the UV–vis absorbance on the size and shape of the NPs, in reference to [33]. 

The shape of NPs is very important in nanotechnology, as it affects the NPs’ physicochemical properties. According to the TEM, the AgNPs were spherical in shape with an average size of 20 nm (±5 nm). Results of FTIR analysis recorded the rapid reduction of silver ions to AgNPs, which might be attributed to the presence of reducing agents such as amino acids, amides, and aromatic rings (polyphenols) in the tea extract. The free amino acids in this extract might have combined with the silver atoms and reduced them to AgNPs. Moreover, the free amino acids were responsible for the stability of the NPs, in accordance with the findings of [34]. The current peaks recorded at 592, 487, and 457 cm^−1^ in the FTIR analysis were attributed to the hexagonal phase of the AgNPs, in agreement with the previous study conducted by [35] on zinc oxide NPs. The DLS analysis revealed that the average size of the AgNPs was 170.6 nm. In accordance with the findings of [19], the size of the AgNPs measured by the TEM assay (20 nm) was lower than the size recorded by the DLS analysis (170.6 nm). This might be attributed to the difference in sample preparation processes (i.e., strong solvation effect) of these methods. Zeta potential analysis demonstrated that AgNPs were negatively charged and dispersed in the medium with a value of −20.06. This negative value confirmed the presence of repulsion among the NPs and proved their colloidal stability, in agreement with the previous results of [17,36]. 

The in vitro antifungal potential of the AgNPs against *M. fructigena* was investigated using the poisoned food technique. A pronounced decrease in the percentages of radial growth of the pathogen was observed, especially upon increasing the dose of AgNPs from 25 to 200 mg/L and increasing the preincubation periods from 0 h to 4 days, in agreement with the previous findings of [37]. The percentage increases in diameters of the inhibition zones of the *M. fructigena* on PDA using the agar well diffusion assay were also increased in a concentration-dependent manner of the AgNPs, compared to treatment with the Nystatin antifungal. These observed results might be attributed to the high intensity at which the AgNPs solution was able to saturate and adhere to the fungal hyphae, which were then disrupted [38]. The recorded MIC of the AgNPs was very low (15 mg/L), which expressed their high antifungal potency even at low concentrations compared to the synthetic antifungal. These results are similar to those of [39], who evaluated the antifungal potential of biogenic AgNPs and a semisynthetic drug (simvastatin) against three toxigenic species of Aspergilli.

In the in vivo assay, the coinoculation of the AgNPs with *M. fructigena* into the wounded apple fruits caused a pronounced reduction in the diameters and weights of the rotting lesions, which was dose and time dependent. This could be attributed to the inhibitory action of AgNPs on the germination of the pathogen conidia and on the developing fungal mycelia. These results are similar to the previous findings of [40]. Compared to the untreated controls, increasing the preincubation period with the AgNPs caused pronounced decrease in the tested rot symptoms. This could be attributed to the saturation of wounds within the fruits with the NPs, thus causing an increase in the inhibitory action of NPs on the germination of the *M. fructigena* conidia. These results of the inhibitory activity of AgNPs are similar to those obtained upon applying the synthetic fungicide, in agreement with [21]. It is worth mentioning that AgNPs did not express any phytotoxic activity, as no blackening or any other apparent disorders were observed on the surface of the treated fruit, compared to the nontreated control fruit.

In accordance with the current results, [24] reported that AgNPs penetrate the fungal cell membrane, causing its perforation and resulting in the leakage of different cell components outside of the cell. Such results confirm that the leakage of proteins and DNA out of the *M. fructigena* conidia might be the possible mode of action of these AgNPs against the pathogen, which led to the recorded inhibition of in vitro and in vivo growth of the pathogen. Moreover, previous results recorded by [41] have confirmed that NPs interfere with the respiratory sequence and block the fungal cell division causing its death.

In this study, the stimulation of HaCaT normal human cell lines treated with AgNPs in the cytotoxicity assay expressed the nontoxicity of the NPs at all tested concentrations and incubation periods. Thus, the treatment of the stored apple fruits with AgNPs would be safe upon human consumption. In accordance with the findings of [25], the treatment of 1BR3 cell lines with AgNPs expressed a minor toxic effect. However, upon applying 15 mg/L of the AgNPs (MIC) and after a 72 h incubation period, no further toxic activity was expressed. On the other hand, the treatment of both cells with the fungicide caused significant reductions in their viability percentages. In agreement with the current findings, [24] recorded the survival of HFB 4 normal human cells of up to 80% when treated with AgNPs at a concentration up to 6 mg/L.

In a future study, the author plans to incorporate the obtained AgNPs with agar to form agar-silver nanoparticles (A-AgNPs) as a simple, economical, and effective method of formulating the AgNPs, in reference to [42]. These A-AgNPs will form a protective layer on the surface of the apple fruits, thus increasing their shelf life. Moreover, such a coating will decrease water loss from these fruits with minimal loss of soluble protein contents, in addition to having a potential antifungal effect. Furthermore, these A-AgNPs could be removed by washing the fruits’ surfaces prior to market shipment. This would reduce the possibility of cytotoxicity or genotoxicity following human ingestion, in the event that AgNPs residue is present on the fruits’ surfaces. Upon application of the A-AgNPs, there would be no direct contact between the nanosilver and the fruits’ surfaces, thus reducing any chances of AgNPs penetration inside the fruits. Accordingly, there would be no phytotoxic effects on the fruit’s quality parameters. In a similar study conducted by [43] on cherry tomatoes, weight loss from these fruits during storage was ascribed to respiration and surface moisture evaporation. Treatment of these tomato fruits with AgNPs formed a dense protective coat on their surfaces [44], and thus reduced water vapor transmittance and transpiration and increased the fruit’s shelf life. These findings thus support the results of the current study. The antifungal potential of AgNPs against *M. fructigena* will be investigated after incubation for longer periods (30–45 days) to simulate the nature of the apple fruit stores. The author’s future perspectives also are to use the high performance liquid chromatography (HPLC) and gas chromatography mass spectrometry (GC-MS) techniques to detect the cytotoxicity of AgNPs. Finally, in the near future, I hope to announce the elimination of using harmful synthetic fungicides and encourages the manipulation of the safe and efficient AgNPs as a nanopreservative in fruit stores worldwide.

## Figures and Tables

**Table 1:** In vitro antifungal potential of AgNPs against *M. fructigena* on (a): PDA, (b): CMA, (c): MEA media using poisoned food technique, at different doses of AgNPs (25–200 mg/L) and different preincubation periods (0 h–4 days).

Media	Percentage (%) Reduction of Radial Growth of *M. fructigena*																	
	25 mg AgNPs/L				50 mg AgNPs/L				100 mg AgNPs/L				200 mg AgNPs/L				Nystatin 100 mg/L	Control
	0 h	1 d	2 d	4 d	0 h	1 d	2 d	4 d	0 h	1 d	2 d	4 d	0 h	1 d	2 d	4 d		
**PDA**	17.9 ^d^ ± 0.45	22.2 ^c^ ± 0.52	25.5 ^b^ ± 0.48	33.1 ^a^ ± 1	32.5 ^c^ ± 0.6	42.9 ^b^ ± 1.3	46.1 ^b^ ± 0.59	64.6 ^a^ ± 0.39	47.2 ^d^ ± 0.72	59.2 ^c^ ± 0.49	64.6 ^b^ ± 0.47	76.6 ^a^ ± 1.5	67.9 ^c^ ± 0.47	82 ^b^ ± 0.61	91.8 ^a^ ± 1.4	96.1 ^a^ ± 0.7	98.3 ^a^ ± 1	0
**CMA**	17.8 ^c^ ± 0.73	21.2 ^b^ ± 0.53	22.3 ^b^ ± 1.2	28 ^a^ ± 0.8	23.5 ^c^ ± 1.5	26.8 ^b^ ± 0.65	29.1 ^b^ ± 0.71	33.6 ^a^ ± 1.4	34.7 ^c^ ± 0.89	40.3 ^ab^ ± 0.79	51.6 ^b^ ± 1.6	62.8 ^a^ ± 0.58	62.8 d ± 1.6	74 ^c^ ± 0.79	84.1 ^b^ ± 0.89	92 ^a^ ± 0.76	97.6 ^a^ ± 1.2	0
**MEA**	17.8 ^c^ ± 0.8	24.6 ^b^ ± 1.6	28 ^b^ ± 0.41	31.3 ^a^ ± 0.76	22.3 ^c^ ± 0.7	24.6 ^c^ ± 0.97	29.1 ^b^ ± 1.4	34.7 ^a^ ± 0.91	31.3 ^d^ ± 1.7	34.7 ^c^ ± 0.92	41.4 ^b^ ± 0.79	48.2 ^a^ ± 1.3	63.9 ^d^ ± 0.93	70.7 ^c^ ± 1.4	77.4 ^b^ ± 0.98	88.6 ^a^ ± 1.4	97.6 ^a^ ± 1.6	0

Where PDA: potato dextrose agar, CMA: corn meal agar, MEA: malt extract agar. Results are means of three replicates of the most accurate assay. Columns with different superscript letters indicate significant differences (*p* ≤ 0.05) based on one-way ANOVA analysis. (±): represents the standard deviations.

**Table 2:** In vitro antifungal efficacy of AgNPs against *M. fructigena* on PDA medium at different doses of AgNPs (25–200 mg/L), using agar well diffusion assay.

% Increase in Diameter of Inhibition Zone				
25 mg AgNPs/L	50 mg AgNPs/L	100 mg AgNPs/L	200 mg AgNPs/L	Nystatin (100 mg/L)
13.6 ^a^ (±0.8)	23.4 ^b^ (±1.2)	46.2 ^c^ (±0.5)	59.1 ^d^ (±1.1)	61.4 ^d^ (±0.7)

Where PDA: potato dextrose agar medium. Results are means of three replicates of the most accurate assay. Columns with different superscript letters indicate significant differences (*p* ≤ 0.05) based on one-way ANOVA analysis. (±): represents the standard deviations.

**Table 3:** The percentages of viability of HaCaT and 1BR3 normal human cell lines after treatment with different concentrations of AgNPs and incubation for different periods compared to the nontreated control cells.

Human Cell Lines	Percentage (%) of Cell Viability										
	24 h			48 h			72 h			Fungicide (50 mg/L)	Control (0.5% DMSO)
	15 mg/L	25 mg/L	50 mg/L	15 mg/L	25 mg/L	50 mg/L	15 mg/L	25 mg/L	50 mg/L		
HaCaT	110 ^a^	112 ^ab^	115 ^b^	120 ^a^	110 ^b^	105 ^c^	100 ^a^	102 ^a^	105 ^b^	26 ^a^	100 ^a^
1BR3	98 ^a^	96 ^b^	95 ^b^	98 ^a^	95 ^b^	80 ^c^	100 ^a^	85 ^b^	70 ^c^	21 ^a^	100 ^a^

Results of human cell viability percentages are means of three replicates of the most accurate assay. Percentages with different superscript letters indicate significant differences (*p* ≤ 0.05) based on one-way ANOVA analysis.

## Data Availability

The data presented in this study are available publicly in the published article.
